# Safe Practice of Y-Site Drug Administration: The Case of Colistin and Parenteral Nutrition

**DOI:** 10.3390/pharmaceutics12030292

**Published:** 2020-03-24

**Authors:** Maciej Stawny, Aleksandra Gostyńska, Malwina Nadolna, Anna Jelińska

**Affiliations:** Department of Pharmaceutical Chemistry, Poznan University of Medical Sciences, 6 Grunwaldzka, 60-780 Poznań, Poland; gostynska.aleksandra@spsk2.pl (A.G.); mnadolna@gmail.com (M.N.); ajelinsk@ump.edu.pl (A.J.)

**Keywords:** medical errors, parenteral nutrition, Y-site administration, drug interaction, colistin

## Abstract

A serious problem in everyday clinical practice is the co-administration of drugs using the same infusion line. Potential complications of co-administration of incompatible drugs include precipitation in the infusion line or central venous catheter leading to its occlusion. Administration of precipitate and large lipid droplets into the venous system may lead to the embolization of capillaries and local or systemic inflammatory reactions, with the consequences of venous thrombosis, chronic venous insufficiency, and even pulmonary embolism. The co-administration of drugs must always be confirmed and clearly defined. The study aimed to determine the interaction between colistin (COL) in the dose used during intermittent hemodialysis and five different ready-to-use PN admixtures (PN) (Kabiven, Smofkabiven, Olimel N9E, Nutriflex Lipid Special, and Nutriflex Omega Special). COL-PN compatibilities were tested by comparing physicochemical properties (pH, zeta potential, lipid emulsion particle size) of COL and PN at three time points: immediately after sample preparation, after ten minutes, and after four hours. No changes in the visual inspection were observed. Both PN without COL and COL-PN samples remained white, homogeneous oil-in-water emulsions with no signs of phase separation, precipitation, or color change. There were no significant changes in pH, and the mean droplet diameter remained below the acceptance limit of 500 nm. The zeta potential and osmolality of COL-PN samples ranged from −21.4 to −7.22 mV and from 567 to 1304 mOsm/kg, respectively. The COL does not influence the physical stability of studied PN admixtures. The co-infusion of COL with Kabiven, Nutriflex Lipid Special, Olimel N9E, Nutriflex Omega Special, and Smofkabiven is possible in the dose used during intermittent hemodialysis.

## 1. Introduction

As malnutrition is one of the predictors of mortality and morbidity in critically ill patients, the importance of maintaining an adequate nutrition status in this population is beyond dispute. Parenteral nutrition (PN) is a treatment option for patients who cannot be fed orally or enterally. Administration of PN admixture lasts from 16 to 24 h. Thus, co-infusion of drug with PN admixture in many cases is unavoidable due to the polypharmacy and a limited number of vascular accesses [1,2]. Administration of certain medications with PN admixture using a Y-site may be a solution. This approach is of particular interest in the case of drugs requiring continuous infusion, such as the colistin (polymyxin E, COL).

COL is a lipopeptide antibiotic with activity against many Gram-negative bacteria. Clinical use of COL decreased in the 1970s due to their potential nephrotoxicity and neurotoxicity. It was re-introduced in clinical practice in the 2000s, as a last resort drug, because of the emerging increase of multidrug-resistant Gram-negative pathogens and the limited number of new antimicrobial agents. It is systemically administrated in the pro-drug form of COL methanesulfonate (CMS), which is hydrolyzed to an active form of colistin that exhibits antibacterial activity. COL in the dose of 1 million IU is administered twice a day as a maintenance dose in patients with intermittent hemodialysis [3,4].

Intravenous therapy in critically ill patients is usually carried out via central veins through multi-lumen central catheters. It often happens that the number of drugs administered exceeds the number of vascular accesses, resulting in co-administration of drugs using the same infusion line. Considering the possibility of co-administration of drugs with PN admixture, the incompatibilities that may affect the stability of the lipid emulsion or deactivate the drug substance should be taken into account. The most common signs of incompatibility of drug–PN admixtures are the formation of a precipitate, pH or color change, and degradation of lipid emulsion manifested by emulsion creaming or coalescence [5,6,7,8,9,10]. Potential complications of co-administration of incompatible drugs include precipitation in the infusion line or central venous catheter leading to its occlusion. Administration of precipitate and large lipid droplets into the venous system may lead to the embolization of capillaries and local or systemic inflammatory reactions, with consequences of venous thrombosis, chronic venous insufficiency, and even pulmonary embolism. The occlusion of central venous catheters is the most common complication, occurring in up to 25% of patients. This situation may result in the loss of the possibility of administration of the drugs and PN by the occluded venous access [11,12].

The common use of COL in the critically ill patients undergoing intermittent hemodialysis and the lack of available compatibility studies with worldwide use ready-to-use PN admixtures prompted us to evaluate the physicochemical stability of COL during simulated Y-site administration with PN admixtures.

## 2. Experimental Section

The compatibility of five different ready-to-use PN admixtures (Kabiven, Smofkabiven, Olimel N9E, Nutriflex Lipid Special, and Nutriflex Omega Special) and COL solution in three different volume ratios of 0.5:1, 1:1, and 1.5:1 was tested. PN admixtures were activated and supplemented in aseptic conditions. According to each PN admixture summary of product characteristics (SPC), the specific trace elements and vitamin preparations were chosen for PN supplementation. Olimel N9E 1500 mL (Olimel; Baxter, Lessines, Belgium), Nutriflex Omega Special 1875 mL (Nutriflex OS; B. Braun Melsungen AG, Melsungen, Germany), and Nutriflex Lipid Special 1875 mL (Nutriflex LS; B. Braun Melsungen AG, Melsungen, Germany) were supplemented with one vial of Cernevit (Baxter, Poland) containing the daily dose of vitamins and one ampoule of Tracutil (B. Braun Melsungen AG, Melsungen, Germany) containing the daily dose of trace elements. Kabiven 1540 mL (Kabiven; Fresenius Kabi AB, Uppsala, Sweden) and Smofkabiven 1477 mL (Smofkabiven; Fresenius Kabi AB, Uppsala, Sweden) were supplemented with one vial of Soluvit N (Fresenius Kabi AB, Uppsala, Sweden), containing the daily dose of water-soluble vitamins, and dissolved in one ampoule of Vitalipid N Adult (Fresenius Kabi AB, Uppsala, Sweden), containing the lipid emulsion, and one ampoule of Addamel N (Fresenius Kabi AB, Uppsala, Sweden), containing the daily dose of trace elements.

The COL drug product (Colistin WZF 1,000,000 IU, WZF, Warsaw, Poland) was reconstituted by dilution with 2 mL of water for injection, and the obtained solution was transferred from the vial to 100 mL ecoflac bottle with normal saline (Natrium Chloratum 0.9%, B. Braun Melsungen AG, Melsungen, Germany). The concentration of COL in prepared infusion was 10,000 IU/mL (0.8 mg/mL of CMS).

PN admixtures and COL solutions were mixed in ratios calculated based on their infusion rates, reproducing daily clinical practice. COL rate was 100 mL/h, simulating a 60 min infusion. Considering the volumes of each PN admixture and the daily clinical practice of PN admixtures infusion time, which lasts 16 to 24 h, the infusion rates were calculated. The minimum and maximum infusion rates of PN admixtures were 62 to 117 mL/h. The comparison of the COL infusion rate with the maximum and minimum PN infusion rates allowed calculation of the volume ratios (*v*/*v*) between the drug and PN admixtures that occur in the infusion line during simultaneous administration using the Y-site, which were about 1.5:1 and 1:1. Additionally, for determining the relations between volume ratios and measured parameters, we also tested a third volume ratio of 0.5:1, which is not clinically relevant.

Samples were prepared by mixing the appropriate volume of supplemented PN admixtures and COL solution in 10 mL plastic vial. Each sample was prepared in triplicate and examined immediately after preparation and after 10 min and 4 h of storage at 25 ± 1 °C. The same parameters were measured for supplemented PN admixtures with vitamins and trace elements, as well as for samples obtained by mixing supplemented PN admixtures with COL in the above-mentioned ratios.

Following the European Pharmacopoeia [13], all PN admixtures were visually assessed for the presence of visible particles or color change. Visual inspection was performed against a black-and-white contrast background by two observers. Following the pharmacopoeial requirements for intravenous lipid emulsions, to consider PN admixtures as compatible with COL, the following criteria must be met: practically free from visible particles; no precipitation can be detected by any of the observers upon visual inspection.

The pH was measured at room temperature using a Mettler Toledo Seven Compact pH/ion S220 pH-meter, and the osmolality was measured at room temperature using a 800CL TridentMed osmometer. In accordance with Bouchoud et al. [5], we set up the acceptance criterion of ΔpH ≤0.2 for the pH change during the study period. The acceptance limit for osmolality changes was set as < 5%. The changes in the pH and osmolality exceeding this value may evidence the acid–base changes in the solution (hydrolysis of ingredients) or precipitation.

The particle size of lipid emulsion and zeta potential (ξ) of PN admixtures were measured at 25 °C using a Zetasizer Nano ZS (Malvern Instruments, Malvern, Great Britain) by dynamic light scattering (DLS) and laser Doppler velocimetry, respectively. The sample preparation, particle size, and zeta potential determination were performed according to the methodology described in our previous work [8]. The results of droplet diameter measurements were presented as MDD (intensity weighted mean droplet diameter), dF1 (the diameter of the particles present in the highest intensity in the first fraction), and Dv100 (the diameter below which 100% of the particles lie). The homogeneity of the samples was determined by the polydispersity index (PDI). All the measurements were performed in triplicate, and the results were expressed as average ± standard deviation. To consider the PN admixtures to be compatible with COL, the size of lipid droplets expressed as intensity-weighted MDD cannot exceed the pharmacopeial limit of 500 nm. This criterion was set for the US Pharmacopeia Method I for the determination of the mean droplet size of lipid injectable emulsions [14]. The measuring apparatus was calibrated before use according to the manufacturer’s instructions. As a positive control, we used PN admixtures without the addition of vitamins and trace elements immediately after activation. The negative control was PN admixtures subjected to stress factors (exposure to 150 °C for 30 min or addition of 0.1 mol/L HCl at 1:1 volume ratio).

The data were analyzed using Statistica 12 software (StatSoft). Two-way analyses of variance (ANOVAs) were used to determine the statistical significance between samples. The a priori level of significance was *p* < 0.05. In the case of a major effect or interaction, significant differences between the COL-PN and PN samples under the study conditions were identified using Tukey’s HSD post hoc tests.

## 3. Results

It was confirmed that the PN admixtures with the addition of a daily dose of vitamins and trace elements meet the requirements for intravenous drugs administered through central veins: pH of PN admixtures ranged from 5.44 to 6.26, osmolality ranged from 1166 to 1911 mOsm/kg, and the MDD of the lipid emulsion ranged from 222.2 to 278.3 nm. In addition, the stability of the oil–water system was determined by measuring the zeta potential. The zeta potential values ranged from −6.46 mV to −15.60 mV.

**Figure 1:**
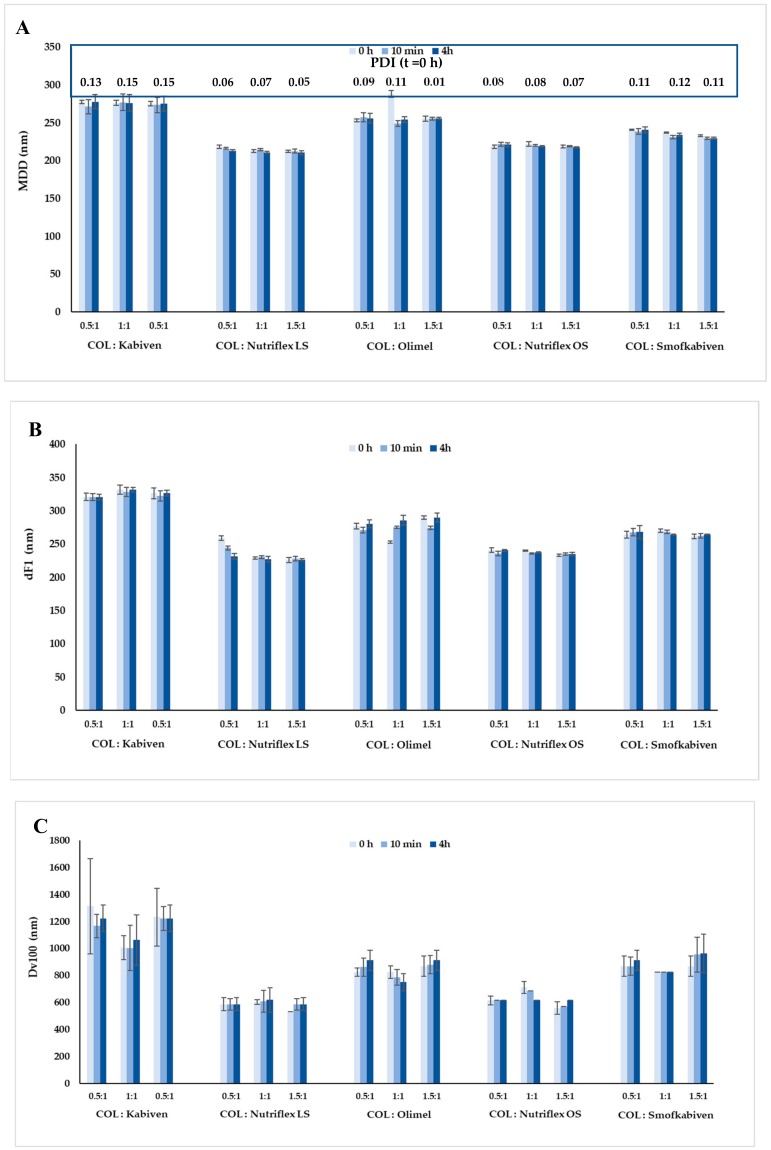
Characterisation of particle size of COL-PN samples: (**A**) MDD; (**B**) dF1, (**C**) Dv100. MDD— intensity weighted mean droplet diameter; dF1—the diameter of the particles present in the highest intensity in the first fraction; Dv100—the diameter below which 100% of the particles lie; PDI— polydispersity index.

**Figure 2:**
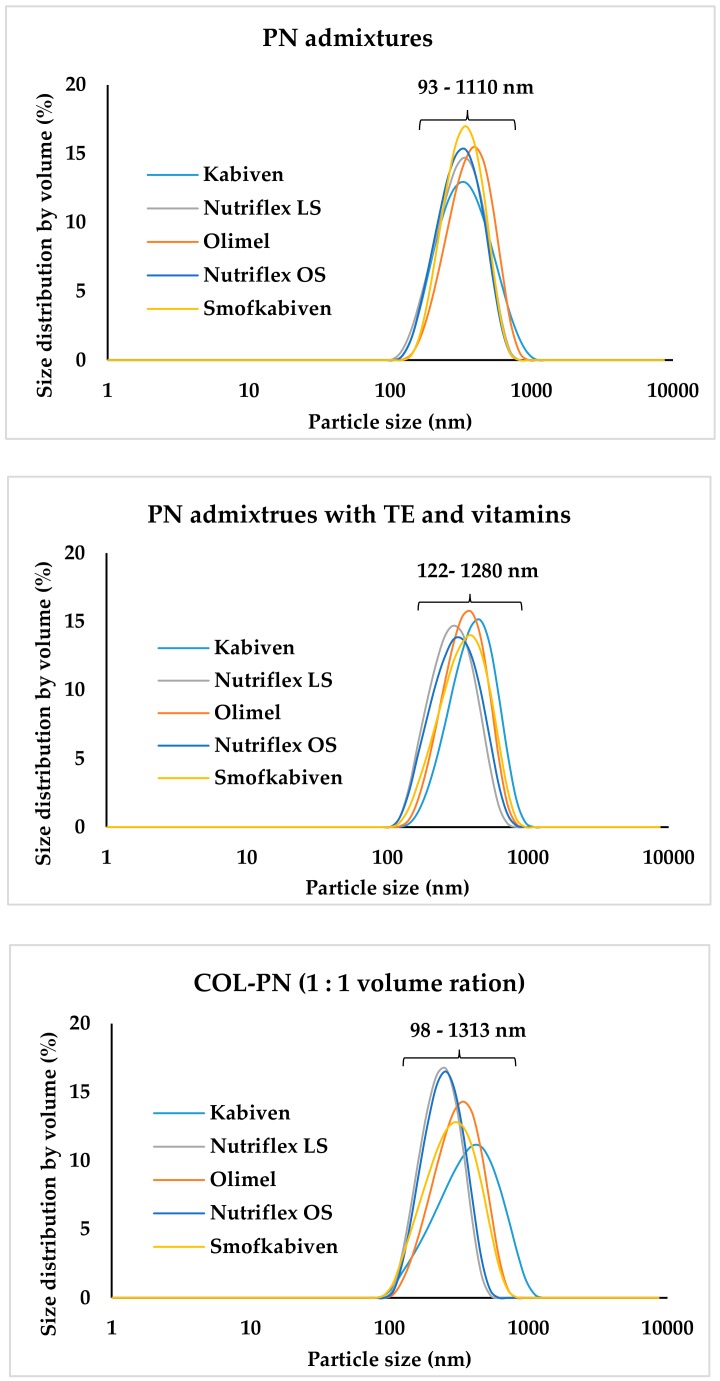
Lipid droplet size distribution by volume for PN admixtures, PN admixtures with TE and vitamins, and COL-PN samples. PN—parenteral nutrition; TE—trace elements.

The pH and osmolality of COL were 7.43 and 279 mOsm/kg, respectively. The pH, osmolality, and zeta potential of COL-PN samples ranged from 5.45 ± 0.01 to 5.32 ± 0.01, 567 ± 5 to 1304 ± 5 mOsm/kg, and −9.23 ± 0.39 to −21.40 ± 0.61 mV, respectively. MDD of COL-PN admixture ranged from 212.33 to 288.07 nm and correlated with COL concentration and the type of lipid emulsion. After four hours of storage, the changes of pH were below 0.04, the changes of osmolality were below 50 mOsm/kg, the changes of zeta potential were ≤5.60 mV, and the changes of MDD were ≤34 nm. The particle size distribution by volume ranged from 93 to 1110 nm, from 122 to 1280 nm, and from 98 to 1313 nm for PN admixtures, PN admixtures with vitamins and trace elements, and for COL-PN samples, respectively. There was no precipitation formation, color change, or any sign of emulsion destabilization. Both PN without COL and COL-PN admixtures remained white, homogeneous oil-in-water emulsions. The results are presented in Table 1 and Figure 1 and Figure 2. The highest values of dF1 were observed for COL-Kabiven samples. However, none of them exceeded the value of 350 nm. Figure 1C represents the highest diameter of any observed droplet in the COL-PN samples, which was the highest for the COL-Kabiven sample in volume ratio 0.5:1 upon preparation (1313 ± 352 nm). In Figure 2, the lipid droplet size distribution by volume and PDI are shown. The results indicate that the addition of vitamins and trace elements slightly affects the distribution of lipid droplet size, in contrast to COL addition, which evidently changes the curve plot as well as PDI value.

## 4. Discussion

For critically ill patients with normal renal function, COL is used in doses ranging from 9 to 12 million IU per day in the treatment of carbapenem-resistant Gram-negative bacteria. In contrast, in patients with acute renal failure undergoing intermittent hemodialysis, the COL dose is reduced and ranges from 0.4 to 2 million IU every 8 h [4]. In our research, we used a dose of 1 million IU of COL, obtaining a COL solution at a concentration of 0.34 mg/mL of COL base (0.8 mg/mL of CMS) and then mixing this solution with PN admixtures to determine the compatibility of such combinations. PN admixtures selected for testing (Kabiven, Smofkabiven, Olimel, Nutriflex LS, and Nutriflex OS) are ready-to-use preparations commonly used in clinical practice all over the world. The caloric value of PN admixtures ranged from 1400 to 2215 kcal, their amino acid content ranged from 51 to 105 g, and their total volume ranged from 1477 to 1875 mL, allowing to meet the energy and nutritional needs of critically ill patients during a short-term nutritional intervention. The theoretical osmolarity of the tested PN admixtures ranged from 1060 to 1545 mOsm/L. The measured parameters (pH, osmolality, zeta potential, and particle size) after adding vitamins and trace elements showed that supplemented PN admixtures were appropriate for administration through central venous access.

To the best of our knowledge, the simultaneous infusion of COL with PN admixtures has not yet been studied. However, the possibility of a combined administration of COL with some antibiotics has been determined [15]. Katip et al. [15] confirmed the compatibility of COL with cefoperazone, sulbactam, ceftazidime, ertapenem, meropenem, imipenem with cilastatin, fosfomycin, linezolid, piperacillin with tazobactam, and vancomycin at concentrations commonly used in intensive care units during simulated Y-site injection.

It should be emphasized that the time of infusion of COL and PN admixture lasts 60 min and 16 to 24 h, respectively. The time of coexistence of both drugs in the infusion line can be counted in minutes. The exact time of contact can be calculated on the basis of the infusion rates of co-infused drugs, the volume of the infusion line, and the placement of Y-site in the infusion line. For our study, we chose three endpoints: immediately after preparation, after 10 min, and after 4 h, in order to fully characterized the potential incompatibilities of studied drugs during infusion. The second endpoint (10 min) was chosen as it is the true-closest value of the longest time of contact of Y-site co-infused drugs in case of the lowest value of infusion rates and the highest volume of infusion line. To calculate this value we assumed that the volume of infusion line is 10 mL. Taking into account the slowest infusion rate (62 mL/h), we calculate that the longest time of contact of the drug with the PN admixture will be about 9 min and 40 s. On the other hand, the shortest time of contact was represented by the first endpoint (immediately after preparation). The third endpoint (four hours) was added to characterize potential interactions in case of any variation of infusion time or the volume of infusion line.

The procedure utilized in this study is in line with the procedures used by other authors. To the best of our knowledge, there are no other endpoints used for the compatibility studies then changes in physicochemical parameters in the appropriate time points. In Y-site compatibility tests performed by Bouchoud et al. [5], three endpoints were chosen: at the time of mixing and after 1 and 4 h. In other studies, inspections were performed during the first 15 min after mixing and at intervals of 1 and 4 h after mixing [16,17,18]. In the compatibility tests carried out by Lee et al. [19], the chosen endpoints were 0, 15, and 30 min after mixing. Staven et al. [20] limited the number of endpoints to two: referred to as 0 h or immediately (described as within 1 h after mixing) and after 4 h. Omotani et al. [9] used multiple endpoints: immediately after preparation and at 1, 3, 6, 9, and 24 h after preparation. Extending the number of endpoints may allow for the observation of time-dependent changes in the studied samples. However, this procedure does not bring data valuable for clinical practice since, as explained above, the actual contact time of two co-infused drugs is definitely shorter.

When considering the compatibility of drugs with PN admixtures and the possibility of their co-administration, not only the invariability of the properties of the drug itself, but also the effect of the drug on the lipid emulsion should be determined. In the course of the study, the characteristics of physicochemical parameters of the PN admixtures without drug and for COL-PN admixture combinations were performed. The results were then compared with each other, as well as with pharmacopoeial limits and recommendations available in the literature. The tested COL-PN admixture combinations remained homogeneous oil-in-water emulsions (their PDI ranged between 0.06 and 0.15 and did not significantly change after 4 h of storage). We did not observe any signs of drug precipitation or signs of oil–water phase separation. The addition of COL to PN admixtures also did not cause significant changes in pH. The measured pH of COL after reconstitution was 7.43, and the addition of COL to PN admixtures did not have a significant influence on the pH of COL-PN admixture samples. This may be due to both the chemical structure and properties of COL as well as the buffering capacity of PN admixture. COL is a peptide antibiotic, consisting of a cyclic heptapeptide with a tripeptide sidechain, and, as an amphipathic drug, COL is characterized by both hydrophobic (due to the fatty acid moiety) and hydrophilic (due to five unmasked gamma-amino groups) properties. COL’s amphipathic properties make the drug dissolve well in both nonpolar lipids and water. The unmasked gamma-amino groups are responsible for the basic character of COL (pKa = 10). During the simultaneous administration of certain drugs with PN admixtures via Y-site, a nonionized form of the drug may precipitate. This situation often applies to drugs that are weak acids or bases and appear in ionized form only at a certain pH. Staven et al. [20] described pH-dependent precipitation of aciclovir, ampicillin, and ondansetron after combination with PN admixtures. In the case of COL, we also checked whether the change in pH of the COL solution after dilution with 0.9% sodium chloride did not cause COL precipitation. We did not observe any precipitation or opalescence of the solution after changing the pH of COL solution to pH 5.00, which further confirmed that COL remains in dissolved form in the pH range from 5.00 to 7.43 (the pH of COL-PN admixtures ranged from 5.45 ± 0.01 to 6.31 ± 0.01). Due to the buffering properties of PN admixtures resulting from the presence of amino acids and acetates, we did not observe significant changes between the difference in pH between pH of PN admixtures without COL and the pH of COL-PN admixtures. COL-PN admixtures had much lower osmolality than PN admixtures, which was due to low COL osmolality (279 ± 2 mOsm/kg). The differences between osmolality measured immediately after sample preparation (*t* = 0 h) and osmolality after 10 min and 4 h of storage were not greater than ± 2%. Only when combining COL with Kabiven in a 1:1 volume ratio, this difference was 6%. Irrespective of the observed osmolality values of COL solution, and COL-PN admixtures, which were below 1000 mOsm/kg, the central venous access must be used during the simultaneous administration of COL and PN admixtures, due to the osmolality of PN admixtures, which was above 1000 mOsm/kg. Administration of drugs characterized by osmolality above 1000 mOsm/kg into peripheral veins may endanger patients’ lives, leading to serious complications resulting from the interaction of the drug with the endothelium and blood cells. The administration of hypertonic solutions to peripheral veins can cause dehydration, contraction of blood cells, and phlebitis. In addition, such administration is associated with pain resulting from blood vessel nociception activation [21].

The zeta potential is a parameter characterizing the stability of the oil-in-water system. In the case of PN admixtures, the lower the zeta potential, the more stable the system. The value of zeta potential depends on both the concentration of electrolytes and on the pH of the PN admixture. Concentrates of lipid emulsion used for PN admixtures preparation are characterized by the zeta potential values in the range from −40 to −50 mV [22]. This is due to the presence of stabilizing phospholipids. The dilution of the lipid emulsion by adding amino acid solutions, water, glucose, and electrolyte solutions reduces its zeta potential absolute value. Especially in the cases of monovalent sodium and potassium ions and bivalent ions of magnesium and calcium, the observed changes are significant. Electrolytes adsorb on the surface of the lipid emulsion particles in a specific and nonspecific way, disturbing the stability of the oil-in-water system. Depending on the composition of PN admixtures, the zeta potential may have values approaching zero [23,24,25,26]. Such admixtures can be still stable for the specific time needed for their administration. Nevertheless, it is necessary to determine the stability of the PN admixture after the addition of any ingredient that may change the charge on the lipid emulsion particles surface. COL, as an anionic compound, caused a concentration-dependent decrease in the value of zeta potential, which further stabilized the oil-in-water system. Four-hour storage of COL-PN admixtures samples resulted in statistically significant changes in zeta potential. The lowest zeta potential values after the addition of COL, and thus the highest oil–water system stability, was observed for Nutriflex LS, which contains equal amounts of long- and medium-chain fatty acids. The observed differences in the zeta potential values for PN admixtures and for COL-PN admixtures samples at t = 0 h and t = 4 h may result from the different compositions of lipid emulsions, different electrolyte compositions, and different amounts of emulsifiers used in preparations. All tested PN admixtures contain egg phospholipids as emulsifiers, but producers in SPC do not provide their content.

The most important parameter used for assessing the safety of the simultaneous administration of PN admixtures and drugs by Y-site is the particle size of the lipid emulsion [27]. Particle size testing uses Method I, recommended by USP chapter 729, which is based on the DLS method and allows the determination of the average lipid emulsion particle size. In accordance with USP requirements, MDD should be below 500 nm [22]. There is an ongoing discussion on the appropriate measurement method for determining the particle size of the lipid emulsion. According to Staven et al. [20], inadequate sensitivity of the Zetasizer nano ZS apparatus may not allow the detection of larger particles of lipid emulsion. Nevertheless, the measuring range of this apparatus is from 0.3 nm to 10 μm, and this method is still recommended by US Pharmacopeia [14] and used by other researchers [28,29,30]. Taking care of the highest quality of the test, to avoid errors related to the suggested lack of appropriate sensitivity of the method used, the particle size measurement was combined with visual control in accordance with the methodology and requirements of the European Pharmacopoeia [13]. Similar to changes in osmolality and zeta potential, a concentration-dependent effect of COL on MDD of lipid emulsion was observed. At the same time, it should be emphasized that MDD of COL-PN admixtures were within the USP requirements, and did not exceed 500 nm. The polydispersity index was below 0.15, and only one fraction of fat emulsion particles was observed for all tested samples.

The physicochemical parameters of COL-PN admixtures are suitable for recommending Y-site co-administration of these drugs. At the same time, it should be emphasized that the co-administration of drugs must always be confirmed and clearly defined. It is estimated that about half of the intravenous drugs prepared in the wards are incorrect [31], of which as much as 25% of incompatibilities in the ICU ward directly threaten the lives of patients [32].

The work presented here demonstrates several limitations. The potential drug incompatibilities were determined only by the evaluation of physicochemical properties. Due to the short time of contact of the drug with the PN admixture in the infusion line during simultaneous administration, we did not determinate the content of the drug. This is in accordance with literature as the chemical stability of the drug, during co-administration using Y-site, is considered to be less important [8,20,33]. Thus, it is not necessary to perform chemical stability assessment in compatibility studies using Y-site.

Secondly, the particle size of lipid emulsion was assessed only using the DLS method and visual assessment. This is considered insufficient according to some authors [34] since the use of Method II [14] and PFAT5 determination is superior by allowing complete characterization of PN admixtures. However, Method I is approved by the US Pharmacopoeia as a suitable measurement approach for particle size determination.

Thirdly, the method used for visible particle detection, described in detail in chapter 2.9.20 of European Pharmacopoeia [13], is based on a statistical process and, in routine practice, the detection limit depends on many factors, such as properties of the particle (morphology and number) and properties of the solution (viscosity, rheology, opalescence, and refractive index). The type of container in terms of size, shape, container material, and fill volume may also affect the particle detection process. Another limitation of such procedure is the lack of specific size cut-off for particles being visible to the human eye. Thus, the detection limit is proportionate to the size of particles and the probability of their detection [35]. Being aware of the limitations of the method, we performed it using two independent observers and followed it by the microscopic evaluation.

Finally, only one concentration of drug was used in the present study. In the case of an administration of a different concentration, repeat testing would have to be carried out. It should be emphasized, however, that in the present study, three COL:PN admixture volume ratios were examined, and the chosen COL dose is appropriate for the treatment of multi-drug resistant Gram-negative bacteria infections in critically ill patients requiring intermittent hemodialysis.

## 5. Conclusions

Co-administration of drugs, including PN admixtures, must always be confirmed experimentally. The drug incompatibilities can be avoided if clinicians’ decisions about drug administration via Y-site will be based on the literature data. The COL-PN admixtures showed no signs of lipid emulsion destabilization nor precipitate formation. MDD was below the pharmacopoeial limit (500 nm). COL reduced the value of zeta potential, which further stabilized the oil–water system. In the light of conducted studies, simultaneous administration of COL (at doses used in critically ill patients who have intermittent hemodialysis) with PN admixtures (Kabiven, Nutriflex Lipid Special, Nutriflex Omega Special, Olimel N9E, and Smofkabiven) is possible and safe.

## Figures and Tables

**Table 1:** The pH, osmolality and zeta potential of colistin–parenteral nutrition (COL-PN) samples.

Sample	COL:PN	pH ± SD	Osmolality ± SD	Zeta Potential ± SD
			(mOsm/kg)	(mV)
		0 h *	0 h → 10 min → 4 h	0 h → 10 min → 4 h
**Kabiven + COL**	0.5:1	5.49 ± 0.01	(807 ± 4) → (855 ± 3) ** → (857 ± 4) **	(−10.87 ± 0.15) → (−10.73 ± 0.25) → (−8.88 ± 0.03) **
	1:1	5.5 ± 0.01	(671 ± 3) → (672 ± 4) → (673 ± 3)	(−12.03 ± 0.55) → (−11.88 ± 0.0.4) → (−9.35 ± 0.46) **
	1.5:1	5.5 ± 0.01	(567 ± 5) → (565 ± 2) → (567 ± 4)	(−12.83 ± 0.59) → (−12.12 ± 0.33) → (−7.81 ± 0.25) **
**Nutriflex LS + COL**	0.5:1	5.45 ± 0.01	(1304 ± 5) → (1298 ± 4) → (1290 ± 7)	(−17.90 ± 0.70) → (−18.02 ± 0.41) → (−18.50 ± 0.89)
	1:1	5.45 ± 0.01	(967 ± 2) → (977 ± 4) → (982 ± 3)	(−19.70 ± 0.36) → (−19.82 ± 0.04) → (−20.03 ± 0.97)
	1.5:1	5.46 ± 0.01	(821 ± 3) → (817 ± 3) → (818 ± 3)	(−21.40 ± 0.61) → (−21.15 ± 0.33) → (−18.60 ± 0.70)
**Olimel + COL**	0.5:1	6.30 ± 0.00	(1096 ± 8) → (1122 ± 6) → (1119 ± 5)	(−11.17 ± 0.21) → (−11.26 ± 0.08) → (−13.83 ± 0.21)
	1:1	6.30 ± 0.00	(831 ± 2) → (855 ± 4) → (853 ± 1)	(−12.23 ± 0.80) → (−12.14 ± 0.24) → (−11.30 ± 0.44)
	1.5:1	6.31 ± 0.01	(712 ± 6) → (728 ± 4) → (731 ± 3)	(−15.87 ± 2.26) → (−15.12 ± 0.72) → (−10.27 ± 0.15) ^**^
**Nutriflex OS + COL**	0.5:1	5.60 ± 0.01	(1299 ± 4) → (1322 ± 3) → (1329 ± 9)	(−9.48 ± 0.27) → (−9.57 ± 0.41) → (−9.65 ± 0.41)
	1:1	5.60 ± 0.01	(964 ± 3) → (976 ± 4) → (975 ± 4)	(−9.58 ± 0.80) → (−9.61 ± 0.28) → (−9.79 ± 0.28)
	1.5:1	5.60 ± 0.01	(818 ± 0) → (807 ± 4) → (804 ± 0)	(−10.24 ± 0.62) → (−9.98 ± 0.38) → (−10.20 ± 0.66)
**Smofkabiven + COL**	0.5:1	5.45 ± 0.01	(1180 ± 4) → (1188 ± 7) → (1197 ± 5)	(−9.23 ± 0.39) → (−9.11 ± 0.07) → (−7.22 ± 0.14) ^**^
	1:1	5.45 ± 0.01	(885 ± 3) → (891 ± 3) → (897 ± 6)	(−10.09 ± 0.53) → (−9.62 ± 0.41) → (−8.59 ± 0.33) ^**^
	1.5:1	5.45 ± 0.00	(758 ± 4) → (755 ± 3) → (756 ± 2)	(−10.63 ± 0.61) → (−10.11 ± 0.12) → (−7.29 ± 0.45) ^**^

* There were no significant changes between pH values upon preparation and 10 min or 4 h later. ** Significant (*p* < 0.05) differences between values of *t* = 4 h and *t* = 0 h.

## References

[1] Kanji S., Lam J., Johanson C., Singh A., Goddard R., Fairbairn J., Lloyd T., Monsour D., Kakal J. (2010). Systematic review of physical and chemical compatibility of commonly used medications administered by continuous infusion in intensive care units. Crit. Care Med..

[2] Trissel L.A. (2013). Handbook on Injectable Drugs.

[3] Kaye K.S., Pogue J.M., Tran T.B., Nation R.L., Li J. (2016). Agents of last resort: Polymyxin resistance. Infect. Dis. Clin. North. Am..

[4] Jacobs M., Grégoire N., Mégarbane B., Gobin P., Balayn D., Marchand S., Mimoz O., Coueta W. (2016). Population pharmacokinetics of colistin methanesulfonate and colistin in critically ill patients with acute renal failure requiring intermittent hemodialysis. Antimicrob. Agents Chemother..

[5] Bouchoud L., Fonzo-Christe C., Klingmüller M., Bonnabry P. (2013). Compatibility of intravenous medications with parenteral nutrition: in vitro evaluation. J. Parenter. Enteral. Nutr..

[6] Staven V., Iqbal H., Wang S., Grønlie I., Tho I. (2016). Physical compatibility of total parenteral nutrition and drugs in Y-site administration to children from neonates to adolescents. J. Pharm. Pharmacol..

[7] Gostyńska A., Stawny M., Dettlaff K., Jelińska A. (2019). The interactions between ciprofloxacin and parenteral nutrition admixtures. Pharmaceutics.

[8] Stawny M., Nadolna M., Jelińska A. (2019). In vitro compatibility studies of vancomycin with ready-to-use parenteral nutrition admixtures for safer clinical practice. Clin. Nutr..

[9] Omotani S., Aoe M., Esaki S., Nagai K., Hatsuda Y., Mukai J., Teramachi H., Myotoku M. (2018). Compatibility of intravenous fat emulsion with antibiotics for secondary piggyback infusion. Ann. Nutr. Metab..

[10] Stawny M., Gostyńska A., Dettlaff K., Jelińska A., Główka E., Ogrodowczyk M. (2019). Effect of Lipid Emulsion on Stability of Ampicillin in Total Parenteral Nutrition. Nutrients.

[11] Benlabed M., Perez M., Gaudy R., Genay S., Lannoy D., Barthelemy C., Odou P., Lebuffe G., Décaudin B. (2019). Clinical implications of intravenous drug incompatibilities in critically ill patients. Anaesth. Crit. Care Pain Med..

[12] Leopoldino R.W.D., Da Costa T.X., Da Costa T.X., Martins R.R., Oliveira A.G. (2018). Potential drug incompatibilities in the neonatal intensive care unit: a network analysis approach. BMC Pharmacol. Toxicol..

[13] European Directorate for Quality in Medicines and Healthcare (EDQM) (2017). 2.9.20. Particulate contamination: Visible particles. European Pharmacopoeia 9.0.

[14] (2015). The United States Pharmacopeia and National Formulary.

[15] Katip W. (2017). Visual compatibility of colistin injection with other antibiotics during simulated Y-site administration. Am. J. Heal. Pharm..

[16] Trissel L.A., Saenz C.A., Ogundele A.B., Ingram D.S. (2004). Physical compatibility of pemetrexed disodium with other drugs during simulated Y-site administration. Am. J. Heal. Pharm..

[17] Chan P., Bishop A., Kupiec T.C., Trissel L.A., Gole D., Jimidar I.M., Vermeersch H. (2008). Compatibility of ceftobiprole medocaril with selected drugs during simulated Y-site administration. Am. J. Heal. Pharm..

[18] Sullivan T., Forest J.-M., LeClair G. (2015). Compatibility of cloxacillin sodium with selected intravenous drugs during simulated y-site administration. Hosp. Pharm..

[19] Lee T.M., Villareal C.L., Meyer L.M. (2019). Y-Site compatibility of intravenous levetiracetam with commonly used critical care medications. Hosp. Pharm..

[20] Staven V., Wang S., Grønlie I., Tho I. (2015). Development and evaluation of a test program for Y-site compatibility testing of total parenteral nutrition and intravenous drugs. Nutr. J..

[21] Wang W. (2015). Tolerability of hypertonic injectables. Int. J. Pharm..

[22] Gonyon T., Carter P.W., Dahlem O., DeNet A.-R., Owen H., Trouilly J.-L. (2008). Container effects on the physicochemical properties of parenteral lipid emulsions. Nutrition.

[23] Washington C. (1996). Stability of lipid emulsions for drug delivery. Adv. Drug Deliv. Rev..

[24] Washington C., Athersuch A., Kynoch D. (1990). The electrokinetic properties of phospholipid stabilized fat emulsions. IV. The effect of glucose and of pH. Int. J. Pharm..

[25] Washington C. (1990). The electrokinetic properties of phospholipid-stabilized fat emulsions. II. Droplet mobility in mixed electrolytes. Int. J. Pharm..

[26] Télessy I., Balogh J., Csempesz F., Szente V., Dredán J., Zelkó R. (2009). Comparison of the physicochemical properties of MCT-containing fat emulsions in total nutrient admixtures. Colloids Surf. B Biointerfaces.

[27] Hippalgaonkar K., Majumdar S., Kansara V. (2010). Injectable lipid emulsions—advancements, opportunities and challenges. AAPS PharmSciTech.

[28] Garcia J., Garg A., Song Y., Fotios A., Andersen C., Garg S. (2018). Compatibility of intravenous ibuprofen with lipids and parenteral nutrition, for use as a continuous infusion. PLoS ONE.

[29] Mediavilla M.M., Molina A., Navarro L., Grau L., Pujol M.D., Cardenete J., Cardona D., Riera P. (2018). Physicochemical Compatibility of Amiodarone with Parenteral Nutrition. J. Parenter. Enter. Nutr..

[30] Riera P., Garrido-Alejos G., Cardenete J., Moliner E., Zapico-Muniz E., Cardona D., Garin N. (2018). Physicochemical stability and sterility of standard parenteral nutrition solutions and simulated y-site admixtures for neonates. Nutr. Clin. Pr..

[31] Taxis K., Barber N. (2003). Ethnographic study of incidence and severity of intravenous drug errors. BMJ.

[32] Tissot E., Cornette C., Limat S., Mourand J.-L., Becker M., Etievent J.-P., Dupond J.-L., Jacquet M., Woronoff-Lemsi M.-C. (2003). Observational study of potential risk factors of medication administration errors. Pharm. World Sci..

[33] Husson E., Crauste-Manciet S., Hadj-Salah E., Séguier J.-C., Brossard D. (2003). Compatibility of parenteral drugs with commercialized total parenteral admixtures during simulated Y-site infusion. Nutr. Clin. Metabol..

[34] Klang M. (2015). PFAT5 and the evolution of lipid admixture stability. J. Parenter. Enter. Nutr..

[35] Mathonet S., Mahler H.-C., Esswein S.T., Mazaheri M., Cash P., Wuchner K., Kallmeyer G., Das T.K., Finkler C., Lennard A. (2016). A biopharmaceutical industry perspective on the control of visible particles in biotechnology derived injectable drug products. PDA J. Pharm. Sci. Technol..

